# *Photorhabdus* symbiotic bacteria drive stronger microbiome restructuring in *Plodia interpunctella* larvae during infection with *Heterorhabditis* nematodes

**DOI:** 10.3389/fcimb.2026.1838162

**Published:** 2026-06-19

**Authors:** Sreeradha Mallick, George Joseph Chakkalakkal, Christa Heryanto, Jasmine D. Alqassar, Arnaud Martin, Vladimir Lažetić, Ioannis Eleftherianos

**Affiliations:** 1 School of Biological Sciences, Institute for Global Food Security, Queen’s University Belfast, Belfast, United Kingdom; 2 Department of Biological Sciences, The George Washington University, Washington, DC, United States; 3 Unit on Chromosome Dynamics, Division of Developmental Biology, Eunice Kennedy Shriver National Institute of Health, Bethesda, MD, United States

**Keywords:** entomopathogenic nematodes, host-pathogen interactions, microbiome, *Plodia interpunctella*, symbiotic bacteria

## Abstract

The insect microbiome can influence host physiology and responses to infection, yet how it changes during interactions with pathogens remains underexplored. The Indianmeal moth, *Plodia interpunctella*, a major global pest of stored food products, can be targeted for biological control using the entomopathogenic nematodes (EPNs) *Heterorhabditis bacteriophora*. Understanding whether *H. bacteriophora* infection alters the *P. interpunctella* larval microbiome is crucial, since changes in microbial diversity, measured by alpha diversity indices (Faith’s Phylogenetic diversity, Observed Amplicon Sequence Variants, Shannon diversity, and Pielou’s evenness), can affect how the infection develops and influence the success of the EPNs as biological control agents. However, the response of the *P. interpunctella* larval microbiome to *H. bacteriophora* infection has not been well-characterized. Here, we investigated how the *P. interpunctella* larval microbiome changes following infection with either symbiotic (carrying the symbiotic bacteria *Photorhabdus luminescens*) or axenic (lacking bacterial symbionts) *H. bacteriophora*. Beta diversity analyses (Bray-Curtis dissimilarity, PERMANOVA) revealed shifts in ASV richness (number of observed amplicon sequence variants) and community evenness in the *P. interpunctella* larvae infected with either symbiotic or axenic nematodes. *P. interpunctella* larvae were sampled at 36h and 60h post-infection for 16s rRNA sequencing (READS/SAMPLE). We analyzed 150 P*. interpunctella* larval microbiomes per time point (60 larvae infected with symbiotic *H. bacteriophora*, 60 larvae infected with axenic *H. bacteriophora*, and 30 uninfected larvae). Illumina paired-end sequencing of 16S rRNA V3-V4 libraries yielded a mean sequencing depth of approximately 3.76 × 10^5 read pairs per sample. The UpSet analyses of shared ASVs across uninfected larvae and larvae infected with either symbiotic or axenic *H. bacteriophora* identified distinct ASVs unique to each infection type. LEfSe analysis further identified differentially expressed taxa observed in the microbiome of larvae infected with either symbiotic or axenic *H. bacteriophora*. Notably, larvae infected with symbiotic *H. bacteriophora* showed the highest number of unique ASVs, indicating that larval microbiome restructuring correlates with the presence of the symbiotic bacteria *P. luminescens*. These results indicate that the bacterial symbiont associated with EPNs is an important driver of host microbiome changes during infection, which may influence infection outcomes and the effectiveness of EPN-based biological control.

## Introduction

Microbiomes are an integral part of animal biology, influencing various aspects of their lives, including immune responses and tolerance to environmental challenges, expansion into new ecological niches, and overall regulation of metabolic pathways (Singh et al., 2021). Insects represent the most taxonomically diverse and ecologically dominant group of arthropods (Engel, 2015). Insects maintain close mutualistic associations with their symbionts that inhabit their tissues. These microbial communities span a wide range of groups, including bacteria, viruses, fungi, and diverse single-celled eukaryotes. The insect-microbe interactions are common and significant because they influence the host’s physiology, ecological roles, and overall fitness (Moreau, 2020; Holt et al., 2024). Previous evidence suggests that several insects, such as the mosquito (Pike et al., 2017), silkworm (Chen et al., 2018), and honey bee (Powell et al., 2014) possess a persistent and distinct microbiome. However, in certain insects, for example, crickets, cockroaches, and wasps, the microbiome is transient and is modulated by the surrounding environment and diet (Kane and Breznak, 1991; Reeson et al., 2003).

Given that insect microbiomes can differ widely, it is important to study how these microbial communities change within specific insect groups. Lepidopteran insects are especially important to study because they include many ecologically and economically important species. Lepidoptera, which is the second-largest insect order (Scoble, 1992), are known for their highly intricate and diverse mutualistic interactions (Ravenscraft et al., 2019). In recent years, the lepidopteran microbiome has drawn increasing scientific interest, with much of the focus shifting toward characterizing its ecological significance (Shao et al., 2024). The Indianmeal moth, *Plodia interpunctella*, is a global lepidopteran pest of stored agricultural food products (Mohandass et al., 2007). To combat the massive economic losses incurred due to the *P. interpunctella* infestation in the warehouses storing cultivated food products, entomopathogenic nematodes (EPNs) such as *Heterorhabditis* spp., have been evaluated as potential biocontrol strategies (Mbata and Shapiro-Ilan, 2005; Ramos-Rodríguez et al., 2007). One of the earlier studies on the *P*. *interpunctella* microbiome investigated the bacterial community across different developmental stages, from eggs through adulthood. The microbial composition was dominated by Proteobacteria, with increasing abundance across development, and Burkholderiaceae was the most abundant bacterial family. Other significant genera included *Delftia*, *Propionibacterium*, *Pseudomonas*, and *Stenotrophomonas* (Mereghetti et al., 2019).

Insects serve as hosts for a diverse range of parasites (Vilmos, 1998). EPNs are a group of ubiquitous natural obligate insect parasites that complete their life cycle within the insect body. Once the host insect is nutritionally depleted, infective juveniles (IJs), the only infectious life stage, exit the cadaver to locate and invade new hosts (Forst et al., 1997). Heterorhabditidae is one of the most well-studied families of EPNs, and the genus *Heterorhabditis* has been established as parasitic nematodes infecting lepidopteran, dipteran, and coleopteran pests (Griffin et al., 1990; Peters, 2013). *Heterorhabditis bacteriophora* carries its symbiotic bacterium, *Photorhabdus luminescens*, which is released after invasion of the insect host. The bacteria then multiply rapidly and contribute to host mortality by secreting toxins, degenerative enzymes, and other virulence factors (Waterfield et al., 2009). Previous work has shown that *P. interpunctella* adults are typically more vulnerable to EPN infection compared to larvae. Pathogenicity varies between the EPN members, with symbiotic *H. bacteriophora* causing low mortality rates in adult *P. interpunctella* (Mbata and Shapiro-Ilan, 2005). However, interactions between *P. interpunctella* larvae and *H. bacteriophora* IJs remain poorly understood. We hypothesize that infection by either symbiotic or axenic *H. bacteriophora*, particularly when the nematodes carry their symbiotic bacterium *P. luminescens*, induces restructuring of the *P. interpunctella* larval microbiome. Such microbiome changes may shape how EPN infections progress and could influence how reliably EPNs function as biological control agents.

Insect-microbiome interactions are increasingly identified as major determinants of insect immunity, host fitness, and susceptibility to parasitic infections (Singh et al., 2021). Reviews of insect gut microbial communities highlight their critical roles in nutrient metabolism, detoxification, and immune regulation, and emphasize that shifts in these communities can alter a host’s susceptibility to pathogens, with direct consequences for agricultural pest control (Al Darwish et al., 2025). Because insect microbiomes influence how hosts respond to pathogens, they are likely to play an important role during infections by EPNs. Importantly, recent work has challenged the traditional monoxenic paradigm of EPN biology, which viewed EPN- symbiotic bacteria pairs as the sole drivers of insect mortality. However (Ogier et al., 2020), demonstrated that *Steinernema* IJs carry a frequently associated microbiota beyond their core symbiont *Xenorhabdus* sp., and that certain members of this broader community, such as *Pseudomonas protegens*, contribute to virulence. This shift toward a pathobiome view underscores that microbiome reconfiguration during infection is not only a marker of host-parasite interaction but also a determinant of biocontrol efficacy. Recognizing the microbiome as a functional component of EPN virulence provides a framework for linking microbial shifts to the effectiveness of biological control interventions in real-world systems. Moreover, recent reviews emphasize that despite the proven efficacy of EPNs in diverse cropping systems, their large-scale success is often constrained by differences in host responses, environmental conditions, and microbial interactions (Kaur et al., 2026). This highlights that understanding how insect microbiomes are modified during EPN infection is essential for providing and improving the consistency of EPN-based pest management. We have previously documented changes in the *Drosophila melanogaster* wild-type larval microbiome when the larvae were challenged with *Steinernema carpocapsae* and *S. hermaphroditum* nematodes (Yau et al., 2025). Additionally, another recent study in *D. melanogaster* revealed that infection with *H. bacteriophora* nematodes containing or lacking their symbiotic bacteria *P. luminescens* can alter the larval microbiome (Mallick et al., 2025). These findings raise the question of whether similar microbiome changes occur in lepidopteran hosts commonly infected by EPNs (Poinar, 1975; Stock et al., 2025). Here we address for the first time the question of whether EPN infection alters the microbiome structure and composition of a natural insect host and major agricultural insect pest, such as *P. interpunctella*, focusing on the role of the EPN’s symbiotic bacteria in this process. Specifically, we examined larval microbiome changes following infection with either symbiotic or axenic *H. bacteriophora*. Understanding these changes in the larval microbial community structure may provide insight into host-parasite interactions and factors that influence the effectiveness of EPN-based control strategies.

## Materials and methods

### Insect stocks

Third instar larvae of *P. interpunctella* were used in all experiments, with rearing following standard procedures (Heryanto et al., 2025). We used *P. interpunctella* larvae because EPNs live in the soil where they target lepidopteran larvae that thrive in moist environments where they feed on roots, decaying matter, or microorganisms (Eleftherianos et al., 2009; Dillman and Sternberg, 2012). Moths of the *bFog* strain (Heryanto et al., 2022a) were reared in vented plastic boxes and fed on a mixed, standardized diet containing 44% (w/w) coarse wheat bran, 5% brewer’s yeast flakes, 9% dextrose, 30% glycerin, 3% canola oil, and 10% distilled water. The culture was maintained in an incubator at 27 °C. Relative humidity was maintained at 70%, with a photoperiod of 16:8 (light:dark). In order to synchronize oviposition, mated emerged adults were anesthetized by CO_2_ narcosis and then transferred to oviposition jars.

### Nematode stocks

A stock of *H. bacteriophora* TT01 nematodes was used for the infection of *P. interpunctella* larvae. For amplifying the *H. bacteriophora* stock, third stage larvae of the greater wax moth *Galleria mellonella* were infected with the nematode IJs in 6 cm Petri dishes (VWR, Radnor, PA). The infected caterpillars were incubated at 25 °C in a 12:12-hour light: dark photoperiodic cycle for 7 days. Then, they were transferred to water traps in Petri dishes filled with sterile water (Kenney et al., 2019). Axenic *H. bacteriophora* IJs were obtained following a previously established protocol (Heryanto et al., 2022a). The axenic status of *H. bacteriophora* was confirmed by homogenizing the nematodes, spreading the lysate on selective media, and observing the absence of *P. luminescens* bacterial colony growth on the plates (Castillo et al., 2012). Before using the nematodes in infection assays, the IJs of both symbiotic and axenic *H. bacteriophora* were surface sterilized in 1% bleach solution and rinsed three times with sterile water to remove the bleach residue (Heryanto et al., 2022b).

### Entomopathogenic nematode infection experiments

Petri dishes (VWR, Radnor, PA) were used for the EPN infection experiments. Each *P*. *interpunctella* larva was exposed to 500 surface-sterilized IJs of either symbiotic or axenic *H. bacteriophora*, suspended in 50 µL of sterile water. Both symbiotic and axenic *H*. *bacteriophora* IJs were transferred with utmost care, ensuring they were uniformly distributed over the insect larval body in order to penetrate the larval cuticle. Every uninfected control larva was treated with 50 μL of sterile water. Each infection assay involved twenty larvae, each infected separately with symbiotic *H. bacteriophora*, twenty larvae infected with axenic *H. bacteriophora*, and ten uninfected control larvae. The petri dishes containing larvae infected with either symbiotic or axenic *H*. *bacteriophora* and those with the uninfected control larvae were sealed with tape, allowing sufficient airflow, and then incubated at 25 °C under a 12:12-hour light: dark photoperiod. Three independent experiments were performed to ensure reproducibility of the results.

### DNA extraction

*Plodia interpunctella* larvae exposed to either symbiotic or axenic *H. bacteriophora* and uninfected control larvae were collected at 36- and 60-hour intervals for microbiome analysis. Five live *P. interpunctella* larvae were pooled and placed into a 1.5 mL Eppendorf tube and immediately stored at -80 °C until further processing. Prior to DNA extraction, all larvae underwent surface sterilization, as previously described (Castillo et al., 2012). Genomic DNA was extracted from whole larvae using the Qiagen DNA extraction kit (Qiagen, Germantown, MD). DNA yield was quantified with a Qubit 4 Fluorometer (Thermo Fisher Scientific, Waltham, MA), ensuring a minimum concentration of 20 ng/µL in a final volume of 100 µL. Sample purity values ranged from 1.9 to 2.06, and DNA concentrations spanned 40–180 ng/µL.

### Library preparation

Samples were prepared using the Zymo Research’s Quick-16S kit with phased primers targeting the V3/V4 regions of the 16S gene. Following clean up and normalization, samples were sequenced on a P1 or P2 600cyc NextSeq2000 Flowcell to generate 2x301bp paired-end (PE) reads. Quality control and adapter trimming were performed with bcl-convert1 (v4.2.4). Primer-dimer sequences identified as PCR artefacts were filtered from the generated FASTQ files based on the following criteria: read length > 150 bp, PolyN strings < 10 sequential Ns, PolyG strings < 150 sequential Gs. All the raw sequence files of this study were submitted to the European Nucleotide Archive (ENA) with the study accession number PRJEB110052.

### Amplicon-based microbiome analysis

Microbiome bioinformatic analysis was performed using QIIME 2 (version 2024.5) for amplicon-based community profiling (Bolyen et al., 2019). Initial pre-processing involved filtering of sequences to retain only those containing v3/v4 primer regions, followed by the removal of these primer sequences using the -q2 cutadapt plugin. Post-trimming, these sequences were denoised by the q2-dada2 plugin to produce high-resolution Amplicon Sequence Variants (ASVs), thus eliminating sequencing errors and chimeras (Callahan et al., 2016). Taxonomic classification was carried out using a Naive Bayes classifier trained on the SILVA 138 reference database (Quast et al., 2013), followed by the removal of ASVs identified as mitochondrial or chloroplast in origin. To mitigate differences in sequencing depth, all samples were rarefied to a uniform read count. Representative sequences (unique ASVs assigned by DADA2) were aligned using MAFFT (Katoh and Standley, 2013) and a phylogenetic tree was constructed using FastTree based on a maximum likelihood approach (Price et al., 2010).

Alpha diversity was quantified using Observed Features, Shannon Index, Pielou’s Evenness, and Faith’s Phylogenetic Diversity (Shannon, 1948; Pielou, 1966; Faith, 1992). Beta diversity was evaluated through Jaccard, Bray–Curtis, and both weighted and unweighted UniFrac distances (Lozupone et al., 2011), visualized via Principal Coordinates Analysis (PCoA). Statistical significance was determined using PERMANOVA (p < 0.05). Downstream statistical analyses and visualizations were conducted in R (version 4.2.3). Distinct taxonomic biomarkers were identified using the Linear Discriminant Analysis Effect Size (LEfSe)(Segata et al., 2011) using the microbiomeMarker R package (v1.4.0) (Cao et al., 2022). Differentially abundant taxa across control, axenic, and symbiotic groups were determined using Kruskal-Wallis tests (p<0.05) and a Linear Discriminant Analysis (LDA) threshold >2.0. To account for the compositional structure of the microbiome data and unequal sequencing depth during differential abundance testing, additional differential abundance analysis was performed using ANCOM-BC2 on non-rarefied ASV count data in R. The statistical model incorporated Treatment, Time, and Treatment x Time interaction to evaluate both treatment and time-dependent microbial responses(Lin & Peddada, 2020). Finally, shared and unique ASVs across the Axenic, Control, and Symbiotic groups were visualized using UpSetR (v1.4.0)(Conway et al., 2017).

## Results

### Differences in alpha diversity indices between the *Plodia interpunctella* microbiome of uninfected larvae and larvae infected with *Heterorhabditis bacteriophora*

**Figure 1:**
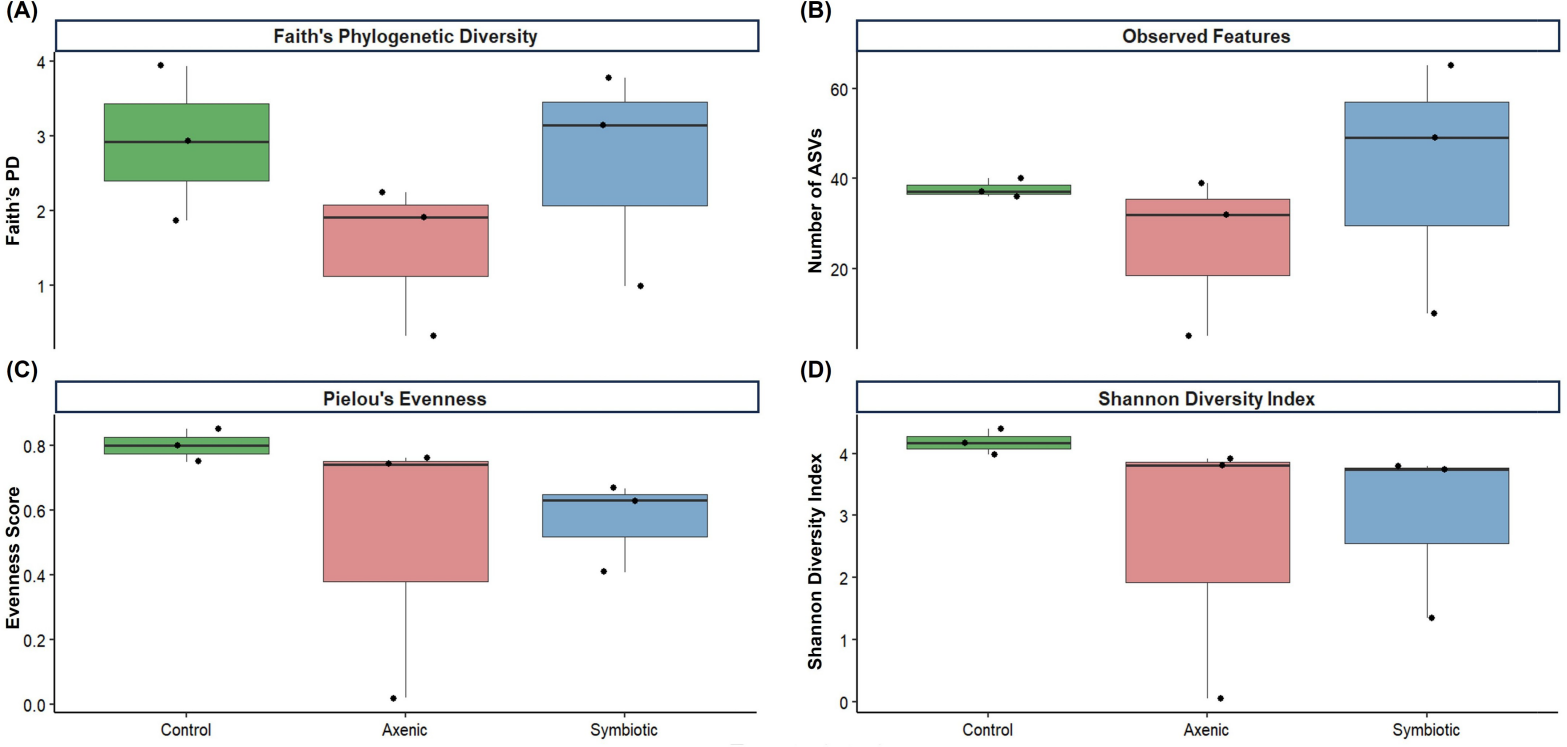
Alpha diversity of microbial communities in uninfected *Plodia interpunctella* larvae **(Control)** and larvae infected with symbiotic or axenic *Heterorhabditis bacteriophora* for 36 hours. Box plots show **(A)** Faith’s phylogenetic diversity, **(B)** Observed Amplicon Sequence Variants (ASVs), **(C)** Pileou’s evenness, and **(D)** Shannon diversity for uninfected *P. interpunctella* larvae (Control) and larvae infected with either symbiotic or axenic *H. bacteriophora*.

To assess within-sample microbial diversity, including species richness and evenness, alpha diversity metrics revealed that larvae infected with axenic *H. bacteriophora* showed the lowest microbial richness and evenness, while larvae infected with symbiotic *H. bacteriophora* exhibited intermediate values. In contrast, uninfected *P. interpunctella* larvae consistently displayed the highest diversity. At 36 hours post-infection, Faith’s phylogenetic diversity, Observed Amplicon Sequence Variants (ASVs), Shannon diversity, and Pielou’s evenness were the lowest in larvae infected with axenic *H. bacteriophora*, intermediate in larvae infected with symbiotic nematodes, and the highest in uninfected larvae (**Figures 1A–D**). These patterns suggest that *H. bacteriophora* infection disturbs the larval microbial community, with the strongest effects occurring when the nematodes lack their symbiotic bacteria *P. luminescens*.

**Figure 2:**
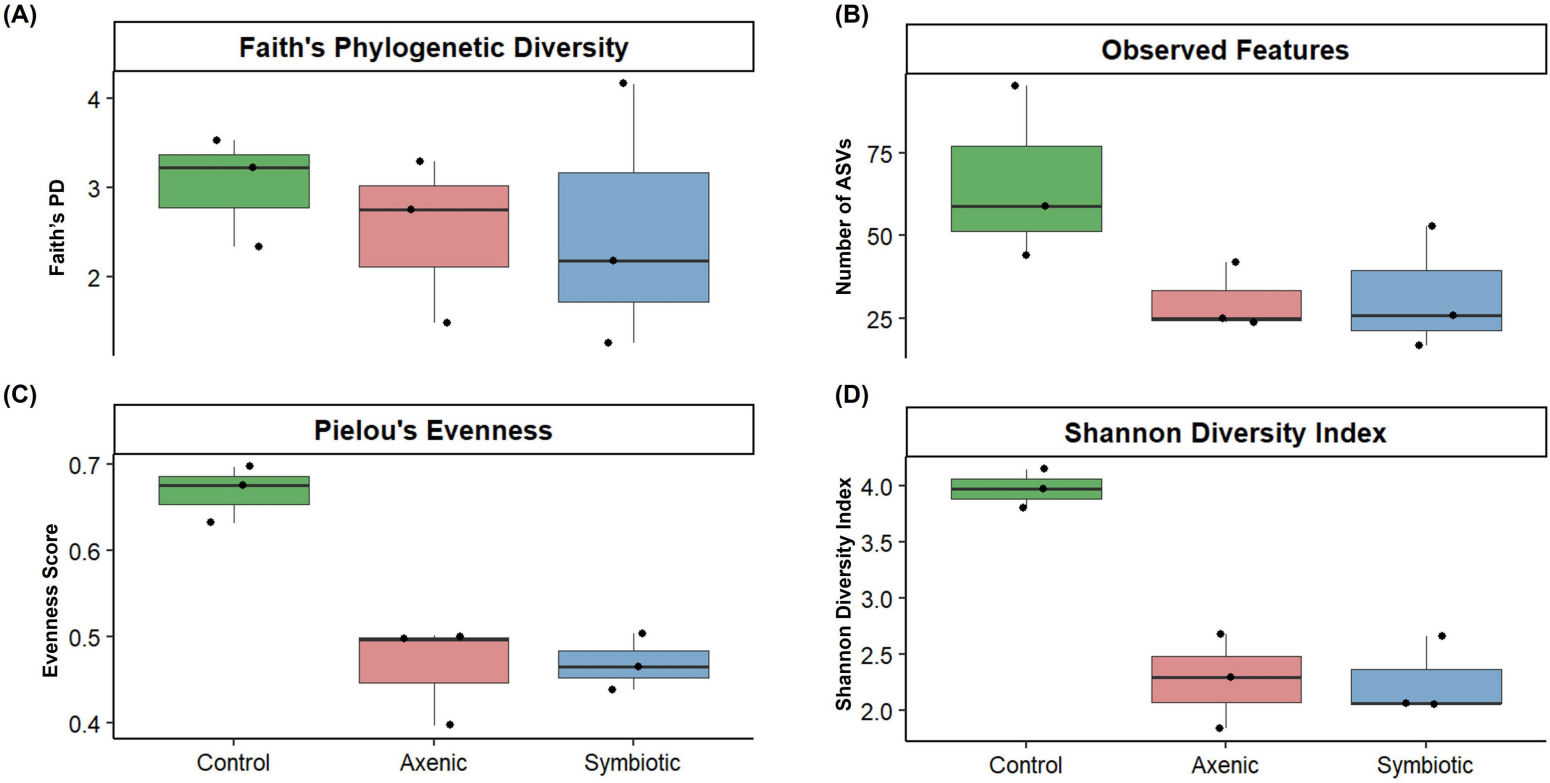
Comparative alpha diversity of the microbial composition in *Plodia interpunctella* larvae infected with either symbiotic or axenic *Heterorhabditis bacteriophora* for 60 hours. Uninfected larvae (Control) sampled at the same time point are shown for comparison. Box plots represent **(A)** Faith’s phylogenetic diversity, **(B)** Observed Amplicon Sequence Variants (ASVs), **(C)** Pileou’s evenness, and **(D)** Shannon diversity for uninfected larvae (Control) and larvae infected with symbiotic or axenic *H. bacteriophora*.

By 60 hours post-infection, the overall microbial diversity remained suppressed in *P. interpunctella* larvae infected with either symbiotic or axenic *H. bacteriophora* compared to the uninfected controls. Faith’s phylogenetic diversity and Observed ASVs continued to show a pronounced decline in larvae infected with axenic *H. bacteriophora*, whereas larvae infected with symbiotic nematodes showed partial recovery or stable conditions across several alpha metrics (**Figures 2A, B**). Similar to the early time point (36 hours), Shannon diversity and Pielou’s evenness remained the lowest in larvae infected with axenic *H. bacteriophora*, intermediate in larvae infected with symbiotic nematodes, and the highest in uninfected larvae (**Figures 2C, D**). Together, these results indicate that the *P. interpunctella* larval microbiome is strongly influenced by the presence or absence of the symbiotic bacterium *P. luminescens*.

### Beta-diversity differences reveal distinct restructuring in *Plodia interpunctella* larval microbiome following nematode infection

**Figure 3:**
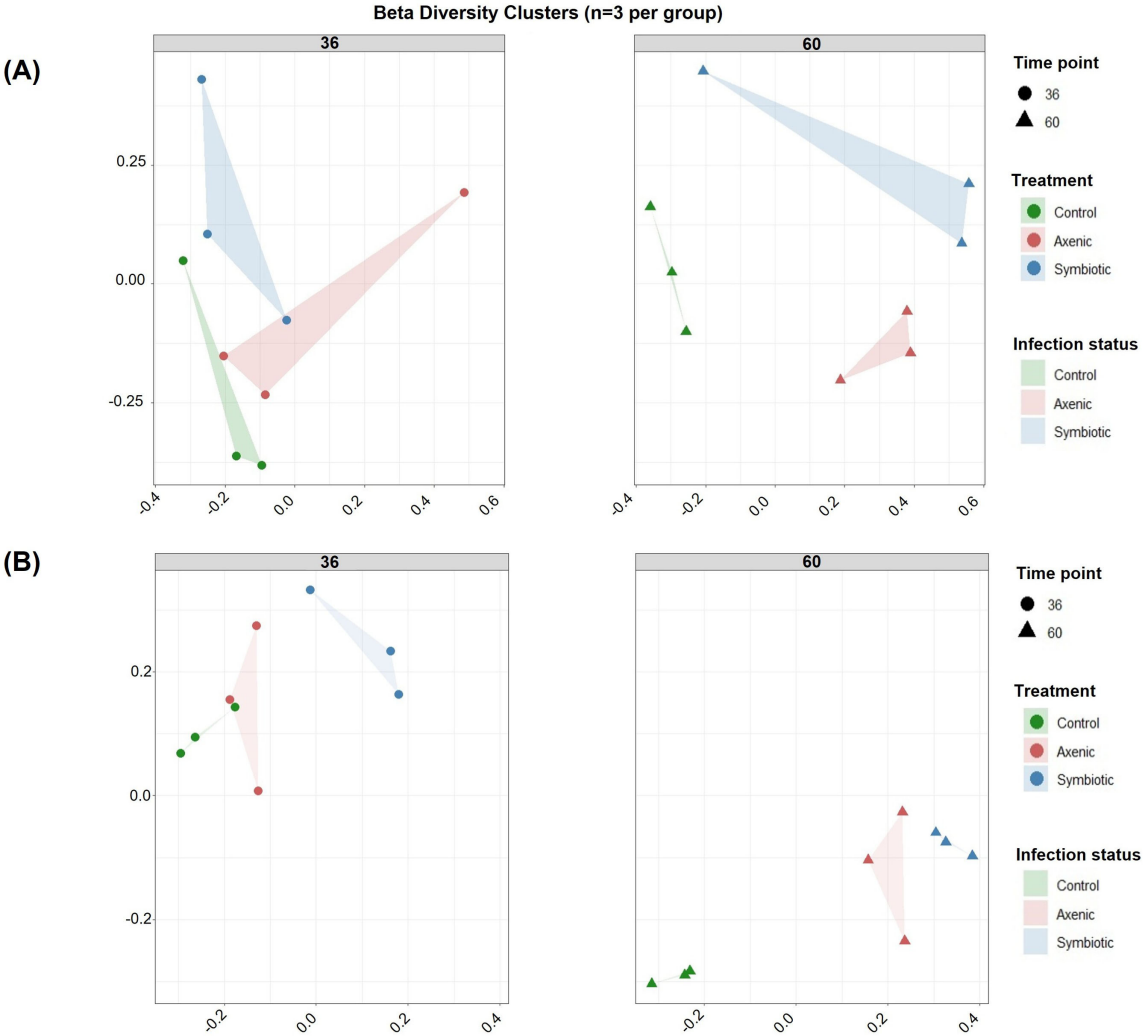
Beta diversity of the microbial composition in *Plodia interpunctella* larvae infected with either symbiotic or axenic *Heterorhabditis bacteriophora* at 36 hours and 60 hours. Uninfected larvae (Control) collected at the same time points are included for comparison. **(A)** Principal coordinate analysis (PCoA) based on the Bray-Curtis dissimilarity, revealing microbiome community structure in uninfected larvae (Control, green) compared to larvae infected with symbiotic *H. bacteriophora* (blue), and larvae infected with axenic *H. bacteriophora* (red). Circles represent 36-hour samples and triangles represent 60-hour samples. Shaded polygons represent group clustering. B) Principal coordinates analysis (PCoA) based on the Jaccard dissimilarity, showing microbial communities spanning across uninfected larvae (Control) and larvae infected with either symbiotic or axenic *H. bacteriophora* at 36 hours (circles) and 60 hours (triangles). Shaded polygons outline samples within each experimental group.

We next compared differences in microbial community composition between samples. At 36 hours, the larval microbiome began forming separate clusters (**Figure 3A**). Microbiome composition of *P. interpunctella* larvae infected with symbiotic *H. bacteriophora* started segregating away from the microbiome of the uninfected control larvae, as well as from the larvae infected with axenic *H. bacteriophora*. This pattern shows that the larval microbiome restructuring had already begun by the 36-hour time-point and was associated with the presence of the symbiotic bacterium *P. luminescens*. This divergence indicated that early larval microbiome changes are influenced by the presence of the symbiotic bacteria. Importantly, the microbiome of the larvae infected with axenic *H. bacteriophora* remained closer to the microbial communities of the uninfected controls, thereby strengthening the argument that there was a weaker dysbiosis in the microbial population in the absence of the *P. luminescens.* By 60 hours, the microbiomes of the three treatment groups (larvae infected with symbiotic nematodes, larvae infected with axenic nematodes, and uninfected larvae) remained clearly separated. However, the larvae infected with symbiotic *H. bacteriophora* showed a more variable and dispersed microbiome structure. In contrast, the microbial communities of larvae infected with axenic *H. bacteriophora* continued to cluster closely and remained intermediate between uninfected controls and larvae infected with symbiotic *H. bacteriophora*. PERMANOVA results supported significant effects of treatment on microbial composition at both time points (p<0.05), with a stronger impact at 60 hours (**Figure 3B**).

### UpSet plot analysis of shared and unique bacterial species in *Plodia interpunctella* larval microbiome during EPN infection

**Figure 4:**
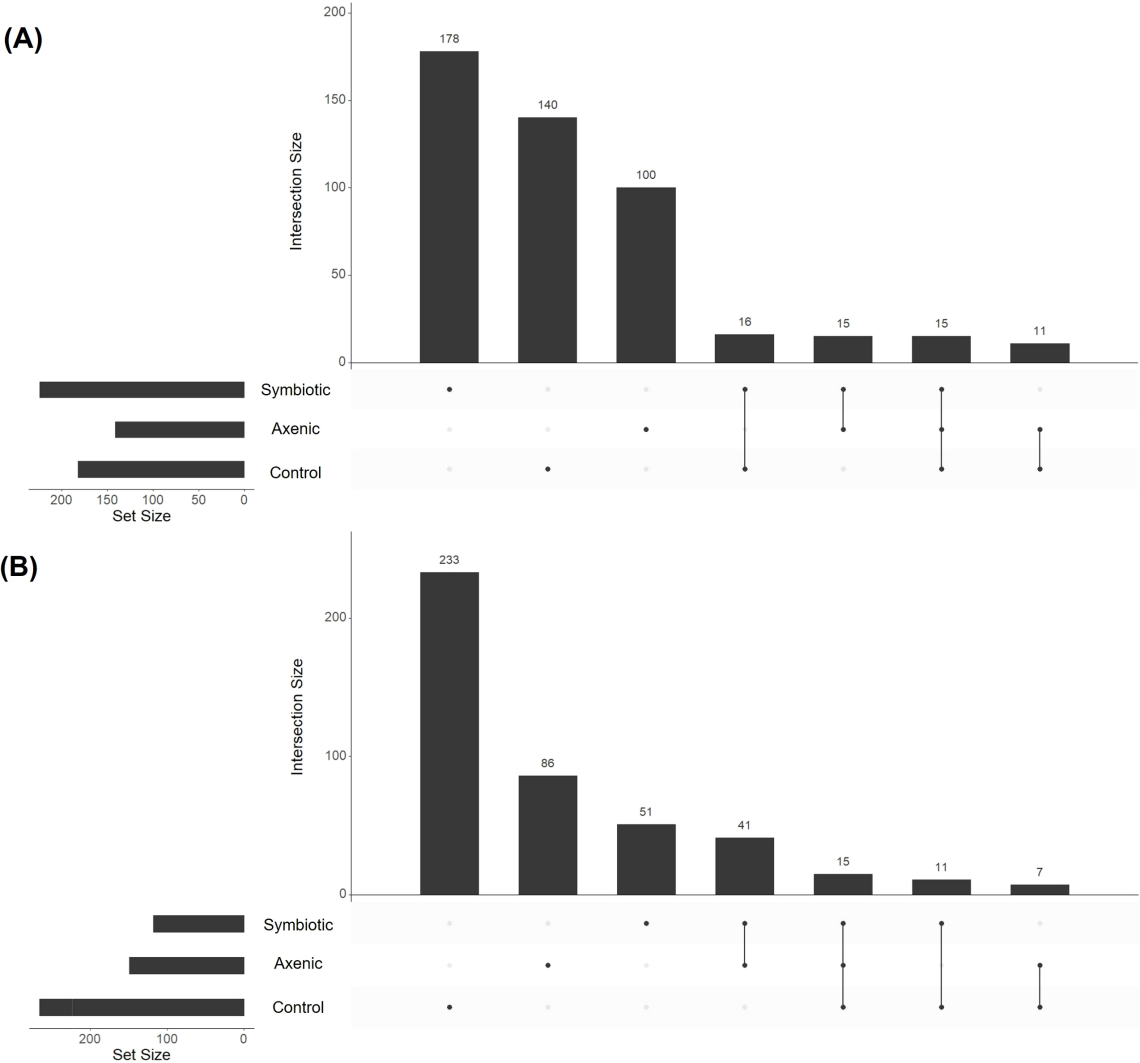
UpSet plots showing amplicon sequence variants (ASVs) dispersion in the microbiome of uninfected *Plodia interpunctella* larvae (Control) and larvae infected with either symbiotic or axenic *Heterorhabditis bacteriophora* at 36 hours and 60 hours. **(A)** and **(B)** The UpSet plots illustrate the unique and overlapping ASVs in uninfected *P. interpunctella* larvae and larvae infected with either symbiotic or axenic nematodes. The plots highlight the ASVs that are exclusive to each experimental condition.

To assess the patterns of shared and unique Amplicon Sequence Variants (ASVs) among the three treatment groups (larvae infected with either symbiotic or axenic *H. bacteriophora* at 36 hours and 60 hours post-infection, and uninfected control larvae), we performed UpSet plot analysis. At 36 hours, a substantial number of unique ASVs were identified in all three treatment groups. Larvae infected with symbiotic *H. bacteriophora* showed the highest number of treatment-specific ASVs (178), followed by the uninfected controls (140), and finally larvae infected with axenic nematodes (100). Sixteen ASVs were shared across the three treatment groups, indicating the presence of a relatively conserved core microbiome at the early time-point. Pairwise overlaps were observed, with 16 ASVs shared between larvae infected with symbiotic *H. bacteriophora* and uninfected controls, 15 between larvae infected with either symbiotic or axenic *H. bacteriophora*, and 11 between uninfected controls and larvae infected with axenic *H. bacteriophora.* Overall, these results suggest that although EPN-associated alterations had begun by 36 hours, bacterial diversity and overlap were still maintained across the different treatments (**Figure 4A**).

By 60 hours, patterns of ASV distribution shifted remarkably. Uninfected control larvae showed the highest number of unique ASVs (233), whereas larvae infected with either symbiotic or axenic *H. bacteriophora* exhibited substantially fewer unique taxa (86 and 51 ASVs, respectively). Notably, the number of ASVs shared across all three treatment groups declined from 16 at 36 hours to 7 at 60 hours, indicating a pronounced reduction in the shared core bacterial community over time. Pairwise overlaps at 60 hours included 41 ASVs shared between symbiotic and axenic *H. bacteriophora* infections, 15 between uninfected control larvae and larvae infected with axenic nematodes, and 11 between uninfected control larvae and larvae infected with symbiotic nematodes (**Figure 4B**).

Collectively, these results demonstrate progressive larval microbiome restructuring following EPN infections. While the bacterial communities remained relatively conserved at 36 hours, substantial divergence was observed by 60 hours, particularly under symbiotic *H. bacteriophora* infection conditions. This was associated with the lowest number of ASVs and reduced overlap with the uninfected controls (**Figures 4A, B**). The presence of the symbiotic bacteria *P. luminescens* within *H. bacteriophora* nematodes appears to be a key driver in reshaping the bacterial community and influencing the host-microbe interactions during infection of *P. interpunctella* larvae.

### The LEFSe analysis identifies differentially expressed taxa in *Plodia interpunctella* larvae infected with *Heterorhabditis bacteriophora*

**Figure 5:**
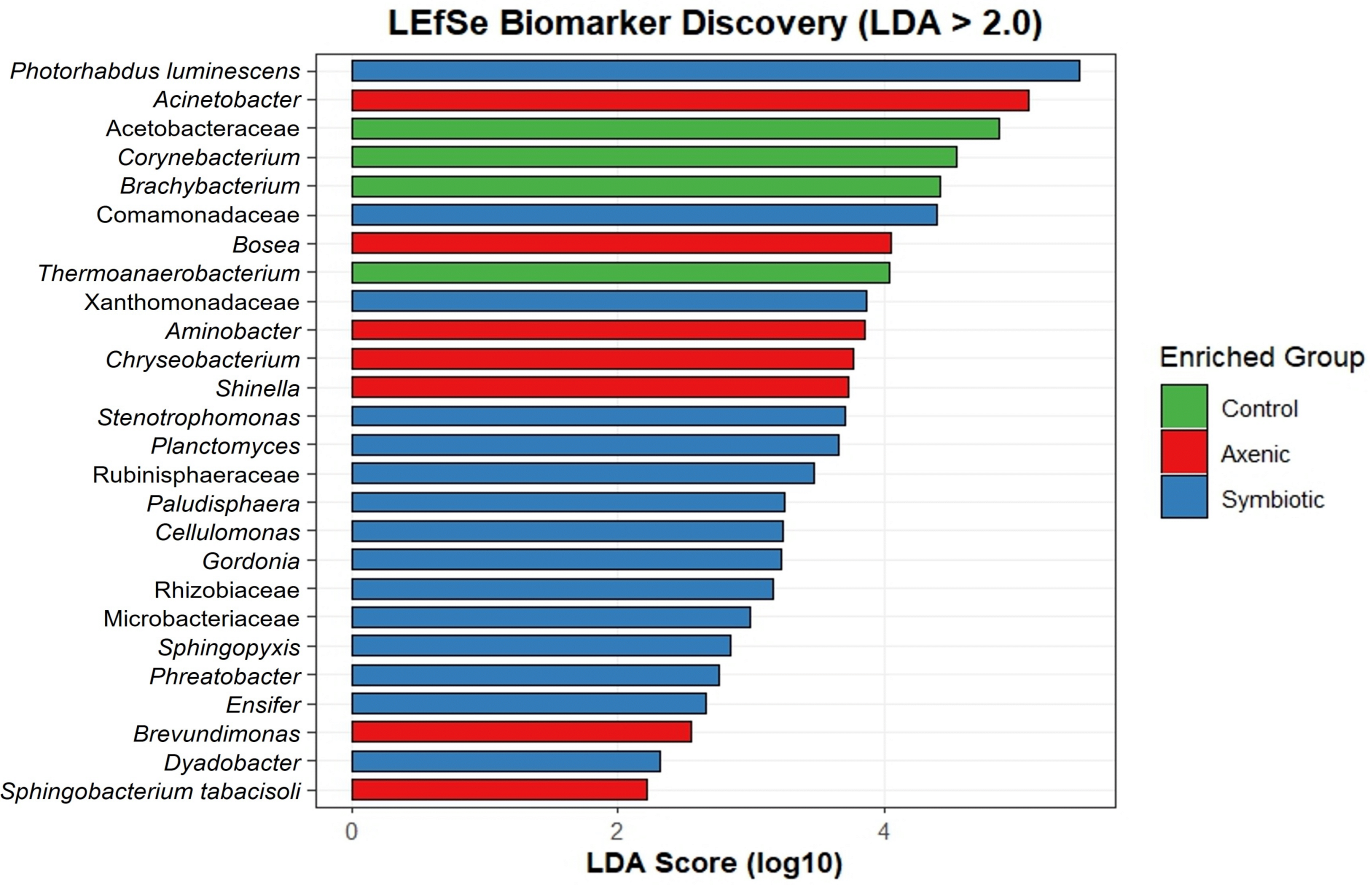
Differential biomarker enrichment in uninfected *Plodia interpunctella* larvae (control) and larvae infected with either symbiotic or axenic *Heterorhabditis bacteriophora*. The bar plot displays the top biomarkers identified by Linear Discriminant Analysis (LDA>2.0). Each taxon is shown with its corresponding LDA score (log 10) indicating the magnitude of enrichment in uninfected larvae (Control) and larvae infected with either symbiotic or axenic nematodes.

To identify distinct bacterial taxa associated with the *P. interpunctella* larvae infected with either symbiotic or axenic *H. bacteriophora* and the uninfected control larvae, we performed LEfSe analysis (LDA >2.0) for samples collected at 36 and 60-hour time points. LDA scores ranging approximately from 2.2 to 5.5, as estimated from the LEfSe analysis (**Figure 5**). Uninfected control larvae were significantly associated with the bacterial groups *Acetobacteraceae*, *Corynebacterium*, *Brachybacterium*, and *Thermoanaerobacterium*. Microbial composition of larvae infected with axenic *H. bacteriophora* was characterized by enrichment of taxa such as *Acinetobacter*, *Bosea*, *Aminobacter, Chryseobacterium, Shinella, Brevundimonas*, and *Sphingobacterium*. In contrast, the microbiome of the larvae infected with symbiotic *H. bacteriophora* included *Stenotrophomonas*, *Planctomyces*, *Paludisphaera*, *Cellulomonas*, *Gordonia*, *Sphingopyxis*, *Phreatobacter*, *Ensifer*, and members of *Comamonadaceae*, *Xanthomonadaceae*, *Rubinisphaeraceae*, *Rhizobiaceae*, and *Microbacteriaceae*. In particular, the microbiome of *P. interpunctella* larvae infected with symbiotic *H. bacteriophora* was mainly dominated by *P. luminescens*, which showed the highest LDA scores. These findings suggest that bacterial community composition differed between symbiotic and axenic *H. bacteriophora* infections. Overall, LEfSe analysis across both 36 and 60 hours demonstrated that symbiotic *H. bacteriophora* carrying *P. luminescens* induced stronger microbiome alterations than the axenic nematodes, indicating that the symbiotic bacteria act as a major driver during EPN infection.

### Heatmap analysis reveals distinct microbiome shifts in the *Plodia interpunctella* larvae infected by either symbiotic or axenic *Heterorhabditis bacteriophora*

**Figure 6:**
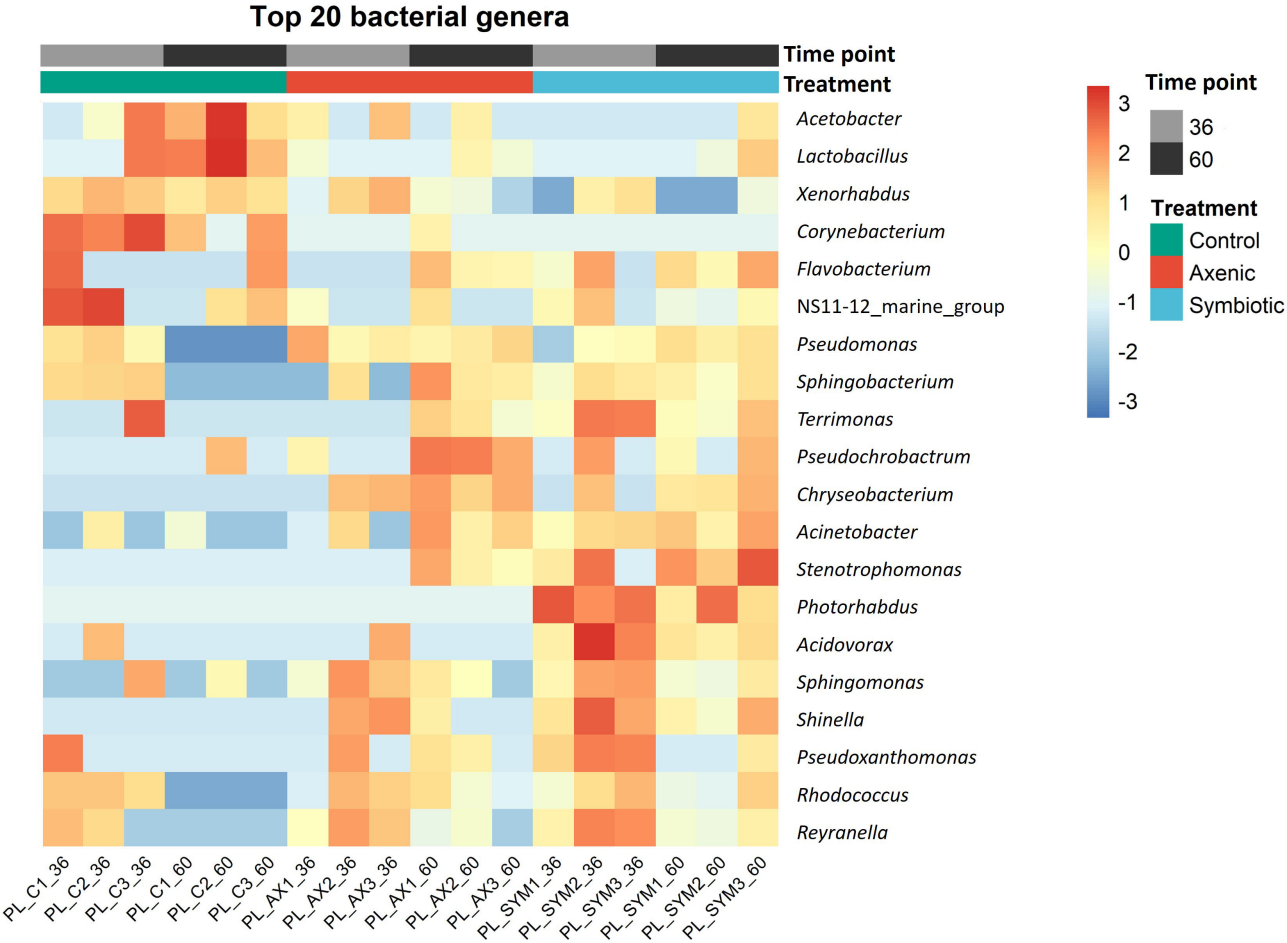
Heatmap of the top 20 bacterial genera in uninfected *Plodia interpunctella* larvae (control) and larvae infected with either symbiotic or axenic *Heterorhabditis bacteriophora*, collected at 36 hours and 60 hours post-infection. Rows represent bacterial genera, and columns correspond to individual biological replicates. The top annotation bars indicate time points and treatment groups. Color intensity reflects scaled relative abundance, with red indicating higher relative abundance and blue indicating lower relative abundance.

Differential abundance analysis using ANCOM-BC2 identified multiple bacterial genera that were significantly different among uninfected control larvae and larvae infected for 36 and 60 hours with either symbiotic or axenic *H. bacteriophora* (**Figure 6**). Significance was assessed using adjusted p-values (FDR <0.05). Hierarchical clustering revealed clear separation by treatments, with additional restructuring by time point. The microbiome changed between 36 and 60 hours within each treatment, but these changes were smaller compared to the differences between the treatment groups. The microbiome of the uninfected control larvae showed abundance of the bacterial genera *Acetobacter*, *Lactobacillus*, and *Corynebacterium*, indicating a diverse bacterial community composition that was distinct from the EPN-infected treatments. In contrast, larvae infected with axenic H. bacteriophora showed an increased relative abundance of genera such as *Pseudomonas*, *Sphingobacterium*, and *Chrysobacterium*. More pronounced shifts were observed in larvae infected with symbiotic *H. bacteriophora* and were strongly associated with higher abundance of *Photorhabdus* along with other genera, including *Stenotrophomonas* and *Sphingomonas*. Overall, these patterns indicate that infection with *H. bacteriophora* leads to substantial microbiome restructuring in *P. interpunctella* larvae, with the presence of *P. luminescens* associated with the most pronounced shifts in community composition.

## Discussion

Here we explored for the first time quantitative and qualitative changes in the microbiome of a natural insect host and important agricultural pest upon EPN infection. Our study demonstrates that *H. bacteriophora* nematodes gradually modified the lepidopteran *P. interpunctella* larval microbiome as infection progressed. At both 36 and 60 hours post infection, alpha diversity metrics consistently showed that axenic *H. bacteriophora* infections caused the strongest decline in the larval microbial richness and evenness. Symbiotic *H. bacteriophora* infections resulted in intermediate microbial diversity reduction compared to the uninfected larvae. However, beta-diversity reveals a distinct clustering of symbiotic *H. bacteriophora* infections while axenic nematode infections showed relatively minor shifts. Although alpha diversity metrics consistently indicated the strongest decline in richness and evenness for larvae infected with axenic nematodes, beta analyses showed greater compositional restructuring in larvae infected with symbiotic nematodes. This apparent discrepancy reflects the fact that alpha and beta diversities capture different ecological dimensions of microbial change. While alpha diversity measures richness and evenness within each sample, beta diversity focuses on differences in the community composition between the samples, which is evident in clustering and ordination analyses. Taken together, these complementary metrics indicate that infection outcomes differ not only in how much larval microbial diversity is lost, but also in how the community changes over time as the infection progresses. These microbiome changes are likely linked to host immune responses, including the activation of antimicrobial peptides and other physiological defenses, which can influence microbial survival and community composition during EPN infection (Stączek et al., 2023; Zhou et al., 2024).

UpSet plots at both time points reveal that *P. interpunctella* larvae infected with symbiotic *H. bacteriophora* harbored the greatest number of unique ASVs, indicating substantial restructuring of the larval microbial community driven by the *H. bacteriophora*-*P. luminescens* association. In contrast, larvae infected with axenic *H. bacteriophora* retained fewer unique taxa and remained closer to the uninfected controls. These results suggest that the nematode alone, without its symbiotic bacteria, is not successful in generating more unique microbial communities in the *P. interpunctella* larval microbiome. The LEfSe analysis further supports this pattern, with the symbiotic nematode infections leading to a broader set of enriched taxa, with the dominant one being *P. luminescens* and multiple bacterial groups which had not been detected in the uninfected controls or in the larvae infected with axenic nematodes. Collectively, our results demonstrate that the *P. interpunctella* larval microbiome is modulated strongly by symbiotic *H. bacteriophora* infections, underscoring the central role of *P*. *luminescens* in forming the insect host bacterial community structure.

Overall fitness and health of the insect host, such as *D. melanogaster*, are significantly impacted by the commensal mutualistic bacteria, *Lactiplantibacillus plantarum* and *Acetobacter pomorum*, forming the fly gut-microbiome (Yun and Hyun, 2023). In the current study, members of the microbial community, including *Acetobacteraceae*, *Corynebacterium*, *Brachybacterium*, and *Thermoanaerobacterium*, were significantly enriched in the uninfected control larvae. These bacterial communities form part of the core larval microbiome and may potentially be involved in the overall maintenance of the *P. interpunctella* larval homeostasis. This is supported by previous studies demonstrating that *Corynebacterium* benefits the cotton bollworm *Helicoverpa armigera*, a major lepidopteran pest (Gu et al., 2022), while *Brachybacterium* enhances fitness of the black cutworm *Agrotis ipsilon*, by enhancing its metabolic performance (ElKraly et al., 2023). In our study, *Acinetobacter* was significantly enriched during axenic *H. bacteriophora* infection, while *Stenotrophomonas* was constantly enriched during the early and late phases of symbiotic *H. bacteriophora* infection. Notably, we have previously reported an increase in *Acinetobacter* and *Stenotrophomonas* in *D. melanogaster* larvae infected with *S. carpocapsae* and *S. hermaphroditum* symbiotic nematodes (Yau et al., 2025). These findings show that *Acinetobacter* (a genus known to be pathogenic to insects) (Ten et al., 2023) and *Stenotrophomonas* behave as an opportunistic taxa that flourish in response to EPN infection-induced host microbiome disruption. Such shifts may reflect modifications in host immune regulation, where disruption of antimicrobial defenses allows opportunistic bacteria to persist and expand (Hanson, 2024; Feng et al., 2025).

Comparisons between the results of the present study and our recent work on the microbiome of *D. melanogaster* larvae infected with symbiotic or axenic *H. bacteriophora* reveal both similarities and notable differences in larval microbiome responses to EPN infection (Mallick et al., 2025). However, these comparisons should be interpreted with caution, as differences in gut physiology, diet, and overall host biology between dipteran and lepidopteran insects may influence microbiome composition and its response to infection. In both insect hosts, EPN infection modifies the larval microbial community structure, demonstrating that *H. bacteriophora* (under symbiotic or axenic conditions) can disrupt the host microbiome. However, the disruption and magnitude of the changes differ considerably. In *D. melanogaster* larvae, alpha- diversity increases following infection, irrespective of symbiotic or axenic nematode infection. However, in *P. interpunctella* larvae, axenic *H. bacteriophora* infection causes a pronounced decline in microbial richness and evenness. Similarly, while axenic *H. bacteriophora* infection in *D. melanogaster* larvae produce several unique ASVs, including the dominant *Stenotrophomonas maltophilia*, the opposite pattern emerges in *P. interpunctella* larvae, where axenic nematode infection leads to a far weaker effect on microbial composition (Mallick et al., 2025). Rather, our present results show that in *P. interpunctella* larvae, *P. luminescens* becomes an important microbiome determinant during symbiotic nematode infection, driving clear beta-diversity shifts and a decline in the number of shared ASVs. Taken together, these comparisons highlight that the impact of EPN infection on larval microbiome varies between insect hosts, reflecting fundamental differences in how each host responds to EPN infection.

Evidence from studies across insects, plants, and vertebrates has consistently established that parasitic nematodes can profoundly impact the structure of the host microbiome. Natural and laboratory infections with the Thelastomatidae nematodes *Leidynema appendiculatam* and *Hammerschmidtiella diesingi* in the cockroaches *Periplaneta fuliginosa* and *Periplaneta americana* have shown that these parasitic nematodes actively restructure the host microbiome (Vicente et al., 2016). Research on plant-parasitic nematodes like the root knot nematode *Meloidogyne javanica* and the root-lesion nematode *Pratylenchus brachyurus* has indicated that the microbial communities moderate the nematode impact and improve plant host performance (Barros et al., 2022). Studies in mammalian systems have further demonstrated that parasitic nematodes can change the host microbiome composition. Infection of *Heligmosomoides polygyrus* in mice modifies the colonic mucus and shifts bacterial abundance (Mules et al., 2024). Additional studies on *Trichuris trichiura* and *Trichuris muris*, infections in humans and mice, respectively, have found that whipworm infections can induce selective modifications in the microbial community structure across different hosts (Rosa et al., 2021). Altogether, these findings emphasize that microbiome modification is a common consequence of nematode infection, strengthening the broader relevance of our observations in *P. interpunctella* larvae. These interactions are closely linked to host physiological changes during infection, including immune activation that can shape microbial community structure (Khan et al., 2023; Haider et al., 2025; Zhang et al., 2025).

## Conclusions and future directions

Our findings provide important insights into how symbiotic and axenic *H. bacteriophora* infections modify the larval microbiome of the economically important stored-food product pest *P. interpunctella*. Including the axenic nematodes in this study allowed us to observe changes in the host larval microbial communities in the absence of the nematode’s natural bacterial partner. This approach provided a clearer picture of how these microbial communities shift following infection with *H. bacteriophora*, both in the presence and absence of *P. luminescens.* Understanding how specific bacterial taxa respond to EPN infection, including the proliferation of opportunistic bacteria, lays out a foundation for predicting how microbiome realignment could impact pest management success. These findings reinforce the importance of considering host-microbiome context when implementing microbial or EPN-based approaches for insect pest control. This information would contribute to both agricultural innovation and a profound understanding of host-microbe-parasite interactions. Importantly, the conclusions in this study are based on observed microbiome changes. Functional assays, including gnotobiotic insect models, microbiome transplantation, or antibiotic treatments, would be needed to confirm these alterations and are planned for future studies.

Future studies could investigate the fundamental basis underlying host-specific microbiome responses to symbiotic and axenic *H. bacteriophora* infections. Especially, assessing how differences in physiology, immune activation, and baseline microbial communities between hosts shape susceptibility to microbiome remodeling will be crucial. Future research could extend microbiome analyses to other economically significant lepidopteran pests, such as the tobacco hornworm *Manduca sexta*, the fall armyworm *Spodoptera frugiperda* and the stem-borer *Chilo suppressalis*, to understand their responses to EPN infection. It would be significant to determine whether manipulation of the pest insect’s microbiome through providing probiotics or specific diets can significantly increase host susceptibility to EPN infection. These insights may lead to novel avenues for improving pest management strategies to protect agricultural products. Moreover, functional assays, including immune gene expression and bacterial colonization experiments targeting dominant taxa such as *Acinetobacter* and *Stenotrophomonas*, could be performed. Metabolomics would further support these analyses by identifying specific host and microbial metabolites that are disrupted or activated during EPN infection. *Acinetobacter baumannii*, associated with the microbiome of the rice pest – the brown planthopper, *Nilaparvata lugens*, has been documented to provide substantial resistance to infection by the entomopathogenic fungus *Metarhizium anisopliae* (Tang et al., 2024). Consistently, Loulou et al., 2023 showed that *Acinetobacter* sp. associated with soil-borne nematodes exhibits entomopathogenic properties. A study has documented that *Spodoptera frugiperda*, an invasive agricultural pest, harbors *Stenotrophomonas maltophilia*, which contributes to the insect’s enhanced resistance to chemical insecticides (Duan et al., 2025). Previous studies have also identified *Stenotrophomonas* spp. on the surface of *Heterorhabditis* spp. IJs, indicating that EPNs may actively transport these bacteria into host insects (Wollenberg et al., 2016). Together, these findings underscore the importance of assessing the roles of these bacteria in insect hosts during EPN infection.

Integrating additional host insects and multiple EPN species in similar studies will further enhance comparative understanding and may lead to the design of more targeted and host-appropriate biocontrol strategies using EPN-bacterial symbiont complexes.

## Data Availability

The datasets presented in this study can be found in online repositories. The names of the repository/repositories and accession number(s) can be found in the article.
